# A *RAPGEF6* variant constitutes a major risk factor for laryngeal paralysis in dogs

**DOI:** 10.1371/journal.pgen.1008416

**Published:** 2019-10-24

**Authors:** Sheida Hadji Rasouliha, Laura Barrientos, Linda Anderegg, Carina Klesty, Jessica Lorenz, Lucie Chevallier, Vidhya Jagannathan, Sarah Rösch, Tosso Leeb

**Affiliations:** 1 Institute of Genetics, Vetsuisse Faculty, University of Bern, Bern, Switzerland; 2 Instituto de Genética Veterinaria (IGEVET), CCT La Plata—CONICET—Facultad de Ciencias Veterinarias, Universidad Nacional de La Plata (UNLP), La Plata, Buenos Aires, Argentina; 3 Tierklinik am Kaiserberg, Duisburg, Germany; 4 Tierklinik Hofheim, Hofheim, Germany; 5 U955 –IMRB, Team 10—Biology of the neuromuscular system, Inserm, UPEC, Ecole nationale vétérinaire d’Alfort, Maisons-Alfort, France; 6 Small Animal Department, ENT-Unit, University of Leipzig, Leipzig, Germany; University of Edinburgh, UNITED KINGDOM

## Abstract

Laryngeal paralysis (LP) is the inability to abduct the arytenoid cartilages during inspiration, resulting in a partial to complete airway obstruction and consequent respiratory distress. Different forms of LP with varying age of onset exist in dogs. Hereditary early onset forms were reported in several dog breeds. In most breeds, hereditary LP is associated with other neurologic pathologies. Using a genome-wide association study and haplotype analyses, we mapped a major genetic risk factor for an early onset LP in Miniature Bull Terriers to a ~1.3 Mb interval on chromosome 11. Whole genome sequencing of an affected Miniature Bull Terrier and comparison to 598 control genomes revealed a 36 bp insertion into exon 15 of the *RAPGEF6* gene (c.1793_1794ins36). The imperfect genotype-phenotype correlation suggested a complex mode of inheritance with a major genetic risk factor involving a recessive risk allele. Homozygosity for the insertion was associated with a 10- to 17-fold increased risk for LP. The insertion allele was only found in Miniature Bull Terriers and Bull Terriers. It was absent from >1000 control dogs of other dog breeds. The insertion sequence contains a splice acceptor motif leading to aberrant splicing in transcripts originating from the mutant allele (r.1732_1780del). This leads to a frameshift and a premature stop codon, p.(Ile587ProfsTer5), removing 64% of the open reading frame. Our results suggest an important role of *RAPGEF6* in laryngeal nerve function and provide new clues to its physiological significance.

## Author summary

Laryngeal paralysis (LP) leads to respiratory distress in affected dogs and can be fatal in severe cases. Many different forms of LP with different etiologies exist. Striking breed predispositions suggest that genetic factors contribute to some forms of LP. During the last years, dog breeders noticed an increased prevalence of an early onset LP in Miniature Bull Terriers. We mapped a major risk locus for this specific form of LP to a ~1.3 Mb interval on dog chromosome 11. Whole genome sequence analysis of an affected dog and comparison to 598 control genomes revealed a single protein-changing genetic variant in the critical interval. This variant, a 36 bp insertion into a coding exon of the *RAPGEF6* gene, creates a cryptic splice site and leads to the expression of an aberrantly spliced transcript with a premature stop codon. This suggests that the insertion results in a loss-of-function allele. Dogs that are homozygous for the insertion have a 10- to 17-fold increased risk to develop LP. The genotype-phenotype association is not perfect, suggesting that other genetic and/or environmental factors also contribute to the development of LP. Our results suggest an important role of *RAPGEF6* in laryngeal nerve function.

## Introduction

Sufficient abduction or opening of the arytenoid cartilages of the larynx during inspiration is essential for breathing [1]. In dogs with laryngeal paralysis (LP), function of one or both recurrent laryngeal nerves is impaired resulting in an insufficient abduction [2]. Therefore, the main clinical sign of dogs with LP is respiratory distress [2–4]. The degree of respiratory distress and the clinical presentation are correlating with the fact of unilateral or bilateral disease and the degree of nerve impairment (paresis versus paralysis) [5]. Clinical signs may vary and include voice impairment (dysphonia), progressive primarily inspiratory laryngeal stridor, exercise intolerance, life-threatening episodes of breathing difficulties, and in cases of bilateral laryngeal disease syncope and cyanosis [2–4].

Non-hereditary and hereditary forms of LP are known [2,6]. Beside traumatic, neoplastic or iatrogenic diseases leading to non-hereditary LP, the most common form of LP is the *geriatric onset laryngeal paralysis polyneuropathy* (GOLPP) in middle-aged and older large and giant breed dogs. GOLPP is supposed to be part of a generalized polyneuropathy [4,7–9]. Hereditary forms of LP in young dogs have been reported in Bull Terriers [10], Bouviers des Flandres [11], Siberian Huskies and Siberian Husky crosses [12,13]. Hereditary LP has been reported to be associated with a juvenile-onset polyneuropathy in Dalmatians [8], Rottweilers [14], white coated German Shepherds [15], American Staffordshire Terriers [16] and in isolated dogs from different breeds [12]. Genetic variants in the *DCNT1* candidate gene were evaluated in Leonbergers and Labrador Retrievers with LP but were not found to be associated with the disease [17]. In Leonbergers, LP and recessive polyneuropathy have been described as a canine homolog of human Charcot-Marie-Tooth neuropathy [18]. More recently, genetic variants in *ARHGEF10* and *GJA9* were shown to cause polyneuropathy in Leonbergers, which includes LP as a clinical sign [19,20].

Diagnosis of LP is based on clinical signs, clinical examination and has to be confirmed by laryngeal endoscopic inspection in anesthesia [21], because clinical signs are not exclusive for LP [4]. Many different underlying causes such as laryngeal tumor or laryngeal collapse can mimic clinical signs of LP.

Recently, a missense variant in *ADAMTS3* was identified in Norwich Terriers with Upper Airway Syndrome. This variant is predisposing to respiratory obstruction due to airway edema within some dog breeds. [22].

**Fig 1:**
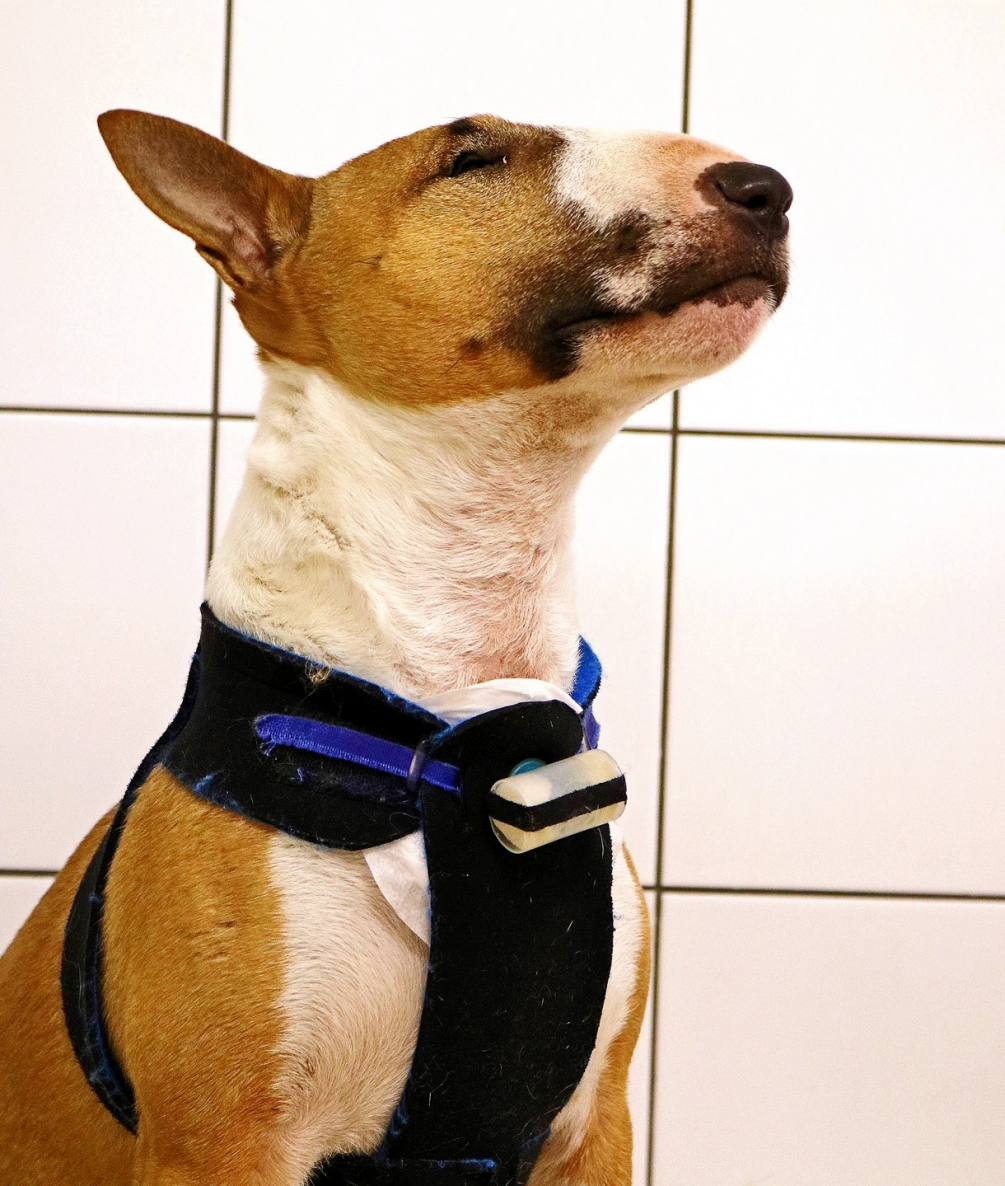
Miniature Bull Terrier affected by laryngeal paralysis (LP). This dog underwent surgery and now carries a permanent tracheostomy tube to alleviate its breathing problems. Tracheostomy is a problematic procedure in dogs as the tracheostoma requires permanent skillful management to avoid infections.

Although not documented in the recent scientific literature, there are anecdotal reports from breeders that an early onset form of LP is common in Miniature Bull Terriers (Fig 1). We therefore initiated this study with the aim of unraveling the underlying genetic cause for LP in Miniature Bull Terriers.

## Results

### Clinical phenotyping

**Fig 2:**
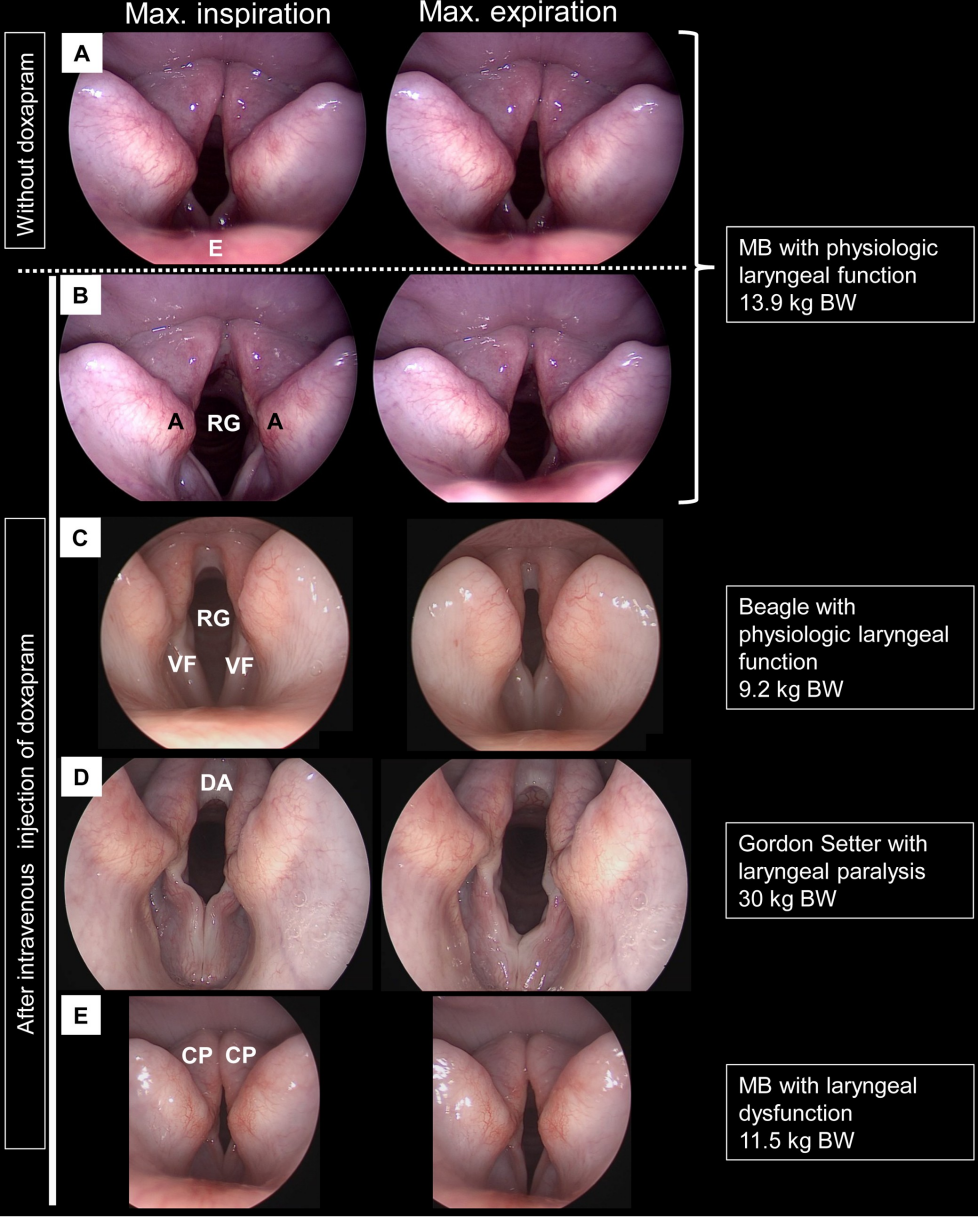
Endoscopic images of the larynx. Frontal view from the oral cavity. E: epiglottis. Black A: arytenoid. RG: *rima glottidis*. VF: vocal fold. DA: dorsal arch of the *rima glottidis*, CP: corniculate process of the arytenoid cartilages. RG is the gap between the two vocal folds. The images in the column “max. inspiration” show the arytenoids at their most abducted/opened position (end of inspiration). Images in the column “max. expiration” are taken at the end of expiration. The arytenoids are supposed to be in their most adducted position. (A, B) Miniature Bull Terrier (MB) with physiologic abduction during inspiration. RG is wider open during inspiration (A, B left column) compared to expiration (A, B right column). Without doxapram (A), the abduction is less pronounced than after stimulation with doxapram (B, immediately after intravenous administration of 1 mg/kg body weight (BW)). (C) Beagle with physiologic laryngeal function. The arytenoids abduct during inspiration and the RG is larger than in expiration. (D) 10-year-old large-breed dog with bilateral LP. In expiration, the vocal cords are not stretched tight anymore and collapsing. During inspiration (left column), arytenoids are not abducted. Due to the negative pressure, both vocal folds are additionally collapsing inward and are reducing the area of the RG. Therefore, the RG is smaller during inspiration than in expiration. (E) Miniature Bull Terrier with LP. No abduction of the arytenoids during inspiration can be observed. The RG is smaller during inspiration than in expiration. Due to the contact of the corniculate processes (CP) of the arytenoid cartilages in in- and expiration and loss of the dorsal arch of the RG, the upper half of the RG is nearly closed. Images S. Rösch.

Laryngoscopy was performed in 36 Miniature Bull Terriers (S1 Table). For none of these dogs, previous trauma or other neurologic diseases had been reported. The examined dogs comprised 33 dogs with breathing problems including inspiratory stridor, dyspnea, exercise intolerance, and in some cases cyanosis and syncope; one dog with cough and syncope without inspiratory stridor or dyspnea and two dogs without breathing problems. In all dogs with inspiratory stridor (n = 33), bilateral LP with abnormal laryngeal function, lacking abduction of the arytenoids during inhalation and/or paradoxical movement of focal folds was diagnosed. The dog with cough and the two dogs without any breathing problems showed physiologic abduction during inhalation (Fig 2).

In contrast to normocephalic dogs with physiologic laryngeal function (Fig 2C) or normocephalic dogs with LP (Fig 2D), the corniculate processes of the arytenoid cartilages in Miniature Bull Terriers appeared to be more closed with loss of the dorsal arch of the *rima glottides* (Fig 2A and 2B). Therefore, in Miniature Bull Terriers with LP smaller *rimae glottidis* in the upper half of the laryngeal inlet were observed than in dogs with GOLPP (Fig 2D and 2E). This fact may explain the severe respiratory distress seen in affected Miniature Bull Terriers.

### Mapping of the LP locus

**Fig 3:**
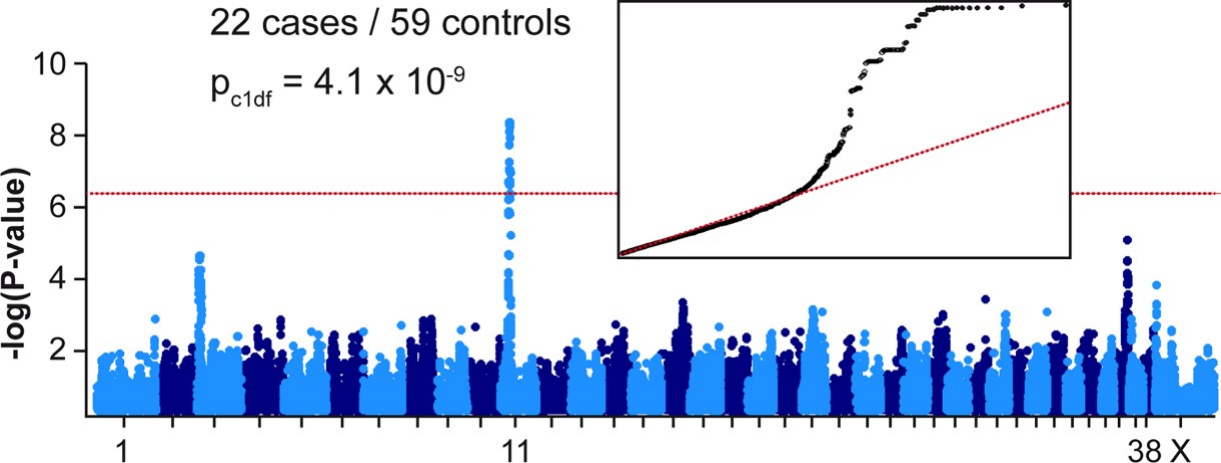
Mapping of the LP locus by GWAS. The Manhattan plot shows a single significant signal at the beginning of chromosome 11. The red line indicates the Bonferroni significance threshold (P_Bonf_ = 4.9 x 10^-7^). The quantile-quantile (QQ) plot in the inset shows the observed versus expected–log(p) values. The straight red line in the QQ plot indicates the distribution of p-values under the null hypothesis. The deviation of p-values at the right side indicates that these markers are stronger associated with the trait than it would be expected by chance.

Due to the striking breed predisposition of Miniature Bull Terriers for a clinically homogenous early onset form of LP, we hypothesized that a new genetic variant might be involved in causing LP in this breed. For the genetic analysis, we performed a genome-wide association study (GWAS) with genotypes from 85 Miniature Bull Terriers. The phenotypes were partly obtained during endoscopic examinations (18 cases / 1 control) and partly based on owners’ reports (5 cases / 61 controls; S1 Table). After quality control, the pruned dataset consisted of 22 LP cases, 59 controls and 102,578 markers. We obtained a single strong association signal with 50 markers exceeding the Bonferroni-corrected genome-wide significance threshold after adjustment for genomic inflation (P_Bonf._ = 4.9 x 10^−7^). All significantly associated markers were located on chromosome 11 within an interval spanning from 18.3 Mb– 20.9 Mb. The most significant SNV was BICF2P1324705 with a p_c1df_-value of 4.1 x 10^−9^ at position 19,230,371 bp on chromosome 11 (Fig 3).

**Fig 4:**
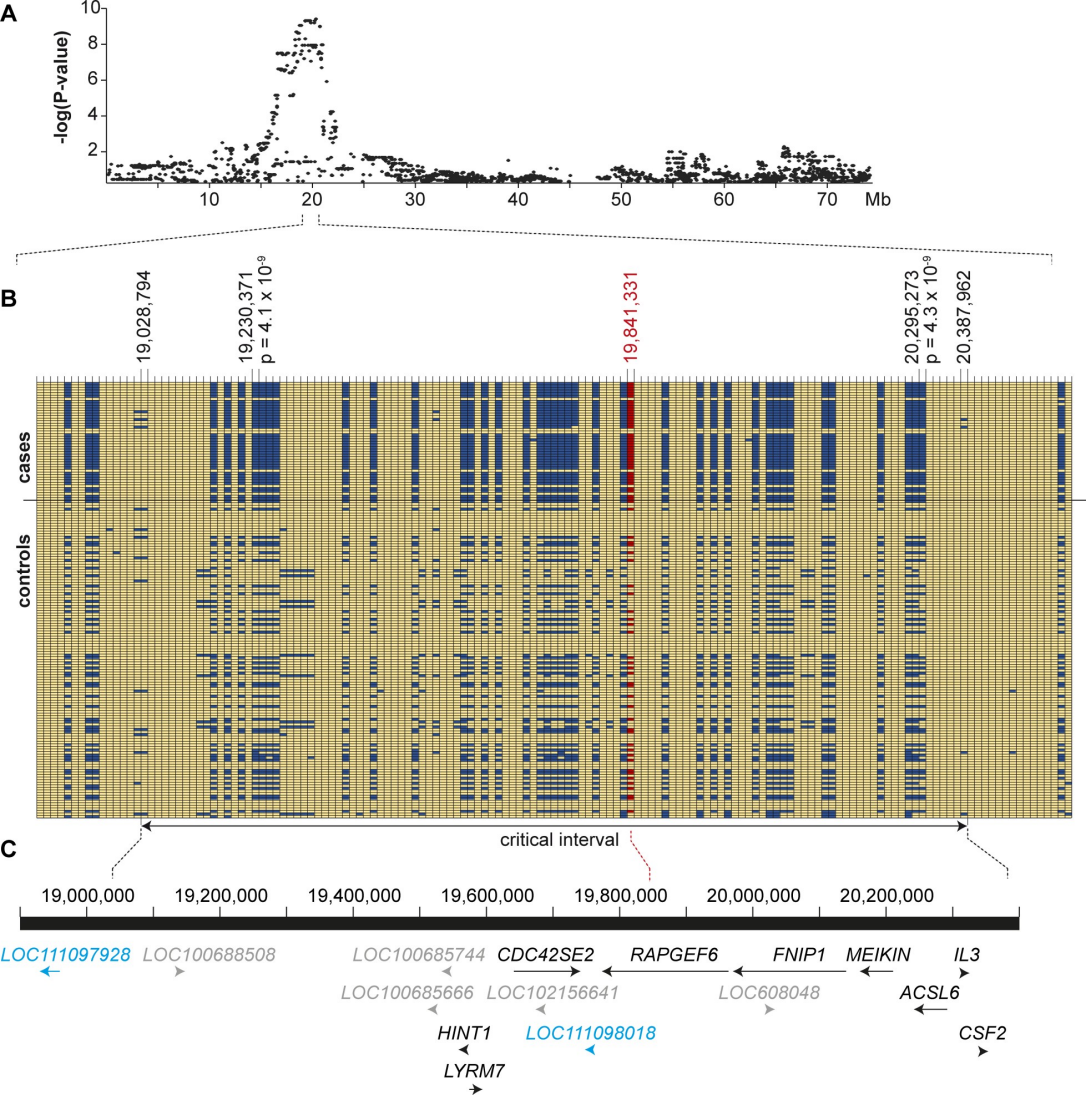
Definition of the critical interval for the LP locus. (A) Details of the GWAS signal on chromosome 11. Markers exceeding the Bonferroni significance threshold were located between 18.3 – 20.9 Mb. **(B)** Haplotype analysis. Seventeen of the tested 22 cases were homozygous for a shared haplotype spanning chr:19,028,794–20,387,962. The positions and p-values of the two best associated markers from the GWAS are indicated. The position of the *RAPGEF6* insertion is indicated in red. The critical interval is indicated at the bottom and defined by recombinations on the left and right side of the shared central haplotype block. **(C)** Gene annotation for the critical interval. The NCBI annotation release 105 listed 9 known protein coding genes (black), 5 computer-predicted protein-coding genes (grey) and one predicted gene for a non-coding RNA (indicated in blue) in the critical interval.

The results of the GWAS indicated a recessive mode of inheritance as most of the cases were homozygous for the risk haplotype. To narrow down the identified region, we visually inspected the phased haplotypes of the cases and performed autozygosity mapping. We searched for homozygous regions with allele sharing and found a region of ~1.4 Mb, which was shared between 17 of the 22 cases (Fig 4). The critical interval for the LP risk variant corresponded to the interval chr11:19,028,794–20,387,962 (CanFam 3.1 assembly).

### Identification of a candidate causative variant

We sequenced the genome of an affected Miniature Bull Terrier at 12.5x coverage and called single nucleotide variants (SNVs) and small indel variants with respect to the CanFam 3.1 reference genome assembly. Automated variant calling identified 2,891,932 homozygous variants in the affected Miniature Bull Terrier. We then compared these variants to whole genome sequence data of 8 wolves and 590 dogs from genetically diverse breeds (other Miniature Bull Terriers and Bull Terriers excluded). We filtered for variants, which were exclusively present in the affected Miniature Bull Terrier. This hard filtering approach reduced the list to 1289 private homozygous variants in the affected dog, but none of them was located in the critical interval (S2 Table, S3 Table).

As the automated variant calling pipeline had not yielded any plausible candidate variants within the critical interval, we visually searched for additional structural variants. Inspection of the short read alignments in the critical interval revealed five additional structural variants with respect to the genome reference sequence in the LP affected dog. Four of them were located in intergenic or intronic regions and not considered to be likely causative for LP. The fifth of these structural variants represented an insertion into exon 15 of the *RAPGEF6* gene after position chr11:19,841,331 (S4 Table).

**Fig 5:**
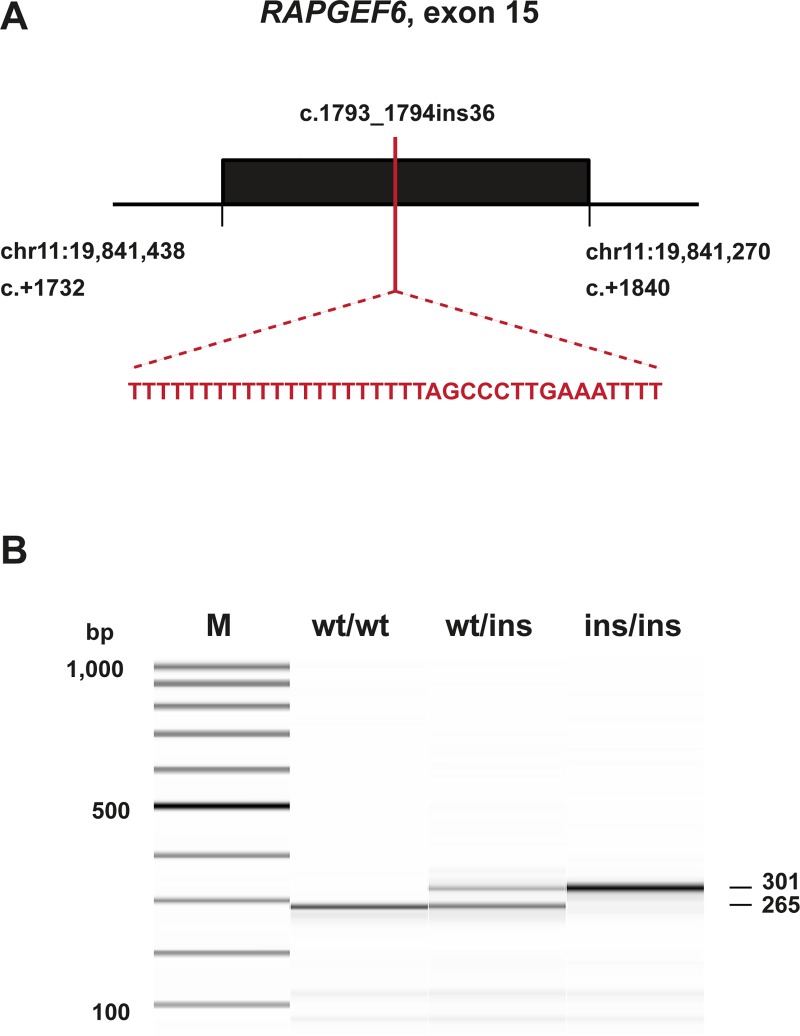
Details of the *RAPGEF6* insertion. **(A)** Schematic representation of exon 15 of the *RAPGEF6* gene. Please note that the orientation is in the direction of transcription and reverse complementary with respect to the genome reference sequence. The insertion consists of a poly-thymine stretch and a duplication of 15 bp of flanking sequence at the integration site. **(B)** Targeted genotyping of the 36 bp insertion by fragment size analysis. PCR products from three dogs with different genotypes were separated on a FragmentAnalyzer capillary gel electrophoresis instrument.

We amplified the *RAPGEF6* exon 15 and flanking sequences by PCR and determined the exact sequence of the insertion by Sanger sequencing. The insertion can be described as XM_846793.5:c.1793_1794insTTTTTTTTTTTTTTTTTTTTTAGCCCTTGAAATTTT, or c.1793_1794ins36 in abbreviated form. It consists of 21 T-residues and the duplication of 15 nucleotides flanking the insertion site (Fig 5).

### Genotype-phenotype association

We genotyped 385 Miniature Bull Terriers, 75 Bull Terriers, and 681 dogs from 73 genetically diverse breeds for the *RAPGEF6* insertion. (S5 Table, S6 Table). Additionally, we visually inspected the short read alignments for the *RAPGEF6* c.1793_1794ins36 variant of 558 dogs from diverse breeds and eight wolves with publicly available genomes (S2 Table). The insertion allele was only found in Miniature Bull Terriers and Bull Terriers but not outside of these breeds. The *RAPGEF6* insertion was not found in 27 additional LP cases from the American Staffordshire Terrier, French Bulldog, Pug, and Staffordshire Bull Terrier breeds (S5 Table).

Most of the LP affected Miniature Bull Terriers were homozygous for the insertion allele (Table 1). However, the data showed an imperfect genotype-phenotype correlation as our study comprised 13 dogs homozygous for the insertion whose owners had not reported any breathing problems (n = 12) or endoscopy revealed no signs for LP (n = 1). On the other hand, there were also 14 dogs with reported breathing problems, which were homozygous wildtype or carried the insertion in heterozygous state. The genotype distribution suggested a complex mode of inheritance involving a major genetic risk factor with recessive mode of inheritance.

**Table 1:** Allele and genotype frequencies at *RAPGEF6*:c.1793_1794ins36 from Miniature Bull Terriers, Bull Terriers and dogs from other breeds.

Breed	Phenotype	n	Allele Freq.		Genotype Frequencies		
			wt	ins	wt/wt	wt/ins	ins/ins
Miniature Bull Terrier	cases (all)	43	0.22	0.78	5 (0.12)	9 (0.21)	29 (0.67)
	cases (endoscopy)	33	0.20	0.80	3 (0.09)	7 (0.21)	23 (0.70)
	controls	200	0.70	0.30	94 (0.47)	93 (0.47)	13 (0.07)
	unknown/excluded^1^	142	0.70	0.30	69 (0.49)	60 (0.42)	13 (0.09)
Bull Terrier	unknown/excluded^1^	75	0.85	0.15	53 (0.71)	22 (0.29)	-
American Staffordshire Terrier	cases	19	1.00	0.00	19 (1.00)	-	-
	controls	9	1.00	0.00	10 (1.00)	-	-
Staffordshire Bull Terrier	cases	4	1.00	0.00	4 (1.00)	-	-
	controls	6	1.00	0.00	6 (1.00)	-	-
Other breeds	unknown	1107^2^	1.00	0.00	1107(1.00)	-	-
Total		1605					

^1^ Exclusion criteria: age of onset ≥ 8 years, cases diagnosed with hypothyroidism, controls < 2 years

^2^ Including 566 genotypes obtained by visual inspection of WGS data in IGV (558 dogs, 8 wolves, S2 Table)

### Relative risk

The relative risk was calculated with a cohort of LP cases endoscopically diagnosed under standardized conditions (n = 21, diagnosed at Tierklinik Hofheim, Tierklinik am Kaiserberg and the University of Leipzig; S1 Table). Miniature Bull Terriers homozygous for the *RAPGEF6* insertion had a 17.2-fold (95% CI: 7.29–40.7, p < 0.0001) increased risk for LP compared to dogs homozygous for the wildtype allele or carrying the mutant allele in heterozygous state. (n = 193).

Relative risk was also calculated with all Miniature Bull Terriers fulfilling the inclusion criteria (n = 243). In this cohort, homozygosity for the *RAPGEF6* insertion was associated with a 9.91-fold (95% CI: 5.75–17.1, p < 0.0001) increased risk for LP compared with dogs, which were homozygous wildtype or carried the insertion allele in heterozygous state. The risk in dogs carrying the insertion in heterozygous state was not significantly increased compared to dogs homozygous for the wildtype allele (RR = 1.75, 95% CI: 0.61–5.03, p = 0.30).

### Functional confirmation

**Fig 6:**
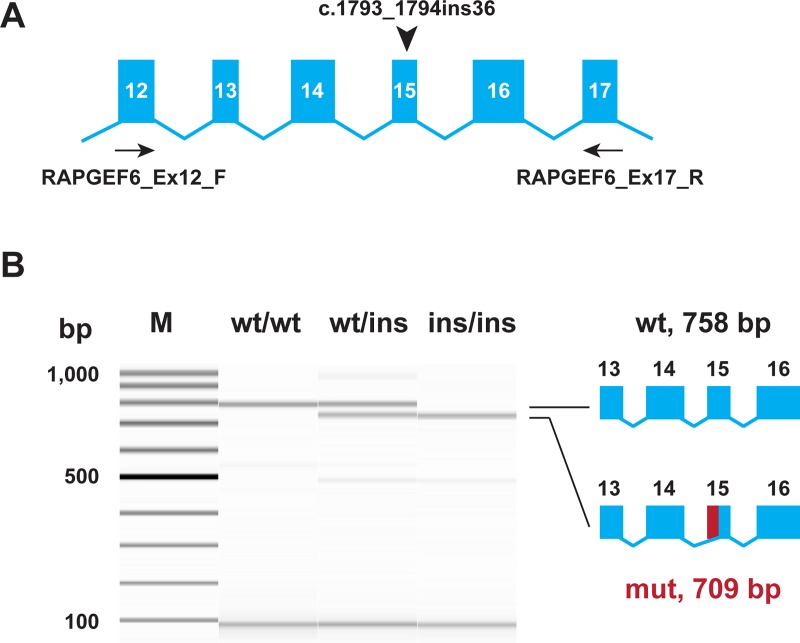
Experimental verification of the *RAPGEF6* splice defect. (A) The genomic organization of the *RAPGEF6* gene in the region of exon 15, position of the insertion, and position of primers for RT-PCR are indicated. (B) RT-PCR was performed with RNA isolated from blood samples of dogs with the three different genotypes. The picture shows a FragmentAnalyzer gel image of the experiment. In the control animal, only the expected 758 bp product is visible. In an LP affected dog homozygous for the insertion, a 709 bp product representing a transcript lacking 49 nucleotides is visible. The genomic insertion leads to the use of an aberrant internal splice acceptor site and a transcript lacking the 5’-end of exon 15 (*RAPGEF6*:r.1732_1780del). The identity of the bands was verified by Sanger sequencing.

For the investigation of the functional consequences of the insertion on the transcript level, we isolated RNA from blood samples of Miniature Bull Terriers with the three different genotypes (*wt/wt*, *wt/ins*, *ins/ins*). RT-PCR with primers located in exon 12 and exon 17 of the *RAPGEF6* gene yielded a band of the expected size in the *wt/wt* dog. The insertion allele gave rise to a transcript lacking 49 nucleotides from the 5’-end of exon 15, XM_846793.5:r.1732_1780del (Fig 6). The formation of the aberrant transcript is due to the presence of a very strong splice acceptor motif created by the insertion, which consists of 25 thymines followed by an AG dinucleotide.

The usage of the aberrant splice site leads to a shift in the open reading frame and an early premature stop codon, XP_851886.2:p.(Ile578ProfsTer5). We did not experimentally verify the expression of the mutant protein. Based on the mutant transcript sequence, 1029 (64%) of the 1606 amino acids of the wildtype RAPGEF6 protein were predicted to be missing in the mutant protein.

### Genotypes at *ADAMTS3*:c.2786G>A (p.Arg929His)

During this study, we additionally genotyped 373 Miniature Bull Terriers, 68 Bull Terriers, 89 French Bulldogs and 12 Pugs for the recently published *ADAMTS3*:c.2786G>A variant in dogs with upper airway syndrome [22]. This mutant allele was not present in Miniature Bull Terriers, Bull Terriers and Pugs. Seventy (79%) French Bulldogs were homozygous for the wildtype allele, 18 (20%) were heterozygous and one dog (1%) was homozygous for the mutant allele. (S1 Table, S5 Table).

## Discussion

In the present study, we identified a 36 bp insertion into exon 15 of the *RAPGEF6* gene as a likely candidate causative variant underlying a major genetic risk factor for an early onset form of LP in Miniature Bull Terriers. The risk locus was unambiguously mapped by GWAS to chromosome 11 and haplotype analyses suggested a critical interval of ~1.4 Mb. The reported insertion was the only phenotype-associated protein-changing variant detected within the critical interval and experiments on the transcript level strongly suggested that the insertion results in a complete inactivation of the *RAPGEF6* gene. Homozygosity for the insertion was associated with a greatly increased risk for LP. Our data were not compatible with a simple Mendelian trait (monogenic autosomal recessive inheritance with full penetrance). Some of the discordant dogs in our study might have been due to erroneous phenotype assignments, as LP can only be reliably diagnosed by endoscopy in living dogs. We had to work with less reliable owner-reported phenotypes in order to obtain the required sample numbers for this study. Heterogeneity and the existence of other clinically similar forms of LP in the breed might explain some of the discordant cases that were not homozygous for the *RAPGEF6* insertion. We verified that *ADAMTS3*:c.2786G>A variant, reported to cause Upper Airway Syndrome in Norwich Terriers [22], does not segregate in Miniature Bull Terriers or Bull Terriers. Being aware of the limitations of our study, we believe that the data nonetheless strongly suggest the causality of the insertion.

The detected 36 bp insertion might have been the result of a retroposon insertion with subsequent deletion of almost the entire retroposon, so that only 15 bp duplicated flanking sequence and 21 bp of poly-A tail remained in the mutant allele [23,24]. Alternatively, we cannot rule out the possibility that the insertion is the result of a non-homologous end-joining repair process, in which the overhanging ends of a spontaneous double-strand break have not been properly processed [25].

*RAPGEF6* encodes the widely expressed Rap guanine nucleotide exchange factor 6. RAPGEF6 and the related RAPGEF2 form a distinct subfamily of guanine nucleotide exchange factors for RAP small GTPases [26–28]. RAPGEF6 is a downstream target of MRAS signaling [27]. RAPGEF6 and RAP signaling have been reported to regulate a wide variety of cellular functions including proliferation, differentiation, and cell adhesion [28]. However, the *in vivo* function of RAPGEF6 is not well understood. *Rapgef6*^*-/-*^ knockout mice are viable and reports on their phenotypes are not entirely consistent. One study reported that *Rapgef6*^*-/-*^ mice were normal in terms of overall growth, appearance and fertility, but had an increased spleen weight [29]. Later on, male infertility was observed in *Rapgef6*^*-/-*^ mice [30]. Finally, a third study reported subtle behavioral alterations and a slightly decreased body weight in *Rapgef6*^*-/-*^ mice [31].

Interestingly, one form of inherited polyneuropathy that also includes LP as a clinical sign in Leonbergers is caused by a genetic variant in *ARHGEF10* encoding Rho guanine nucleotide exchange factor 10 [19]. RAPGEF6 and ARHGEF10 thus both represent guanine nucleotide exchange factors that appear indispensable for the proper function of peripheral neurons with long axons. In our study, the phenotype of *RAPGEF6*^*-/-*^ dogs seemed to be restricted to changes in laryngeal innervation with an early age of onset. *ARHGEF10*^*-/-*^ dogs have a later age of onset of clinical signs, but their phenotype comprises a broader variety of peripheral nerves. It has been shown that a loss of ARHGEF10 leads to defects in axon ensheathment and myelination [18,19].

Inherited forms of LP have been observed in at least three different human families. Autosomal dominant inheritance was postulated, but the causative genetic variants remain unknown [32–34]. Further studies are required to clarify whether *RAPGEF6* variants might be functionally involved in these or similar cases of human LP.

The *RAPGEF6*:c.1793_1794ins36 variant segregates in Miniature Bull Terriers and also Bull Terriers. Miniature Bull Terriers were derived from Bull Terriers (see Material and Methods for further details on breed history). The relatively high frequency of the disease associated allele in the two breeds represents a major threat to the breeding programs. We recommend the introduction of genetic testing and a targeted breeding program to decrease the prevalence of LP. Future matings should be planned with at least one of the breeding animals being clear (*wt/wt*) to avoid the birth of further homozygous mutant offspring. At the same time, it is important to stress that carriers and even homozygous mutant animals should not be immediately excluded from breeding. We recommend to aim at a gradual reduction of the mutant allele. An abrupt exclusion of all carrier animals from breeding would lead to a substantial loss of genetic diversity in the breed and a further increase in inbreeding. This in turn is likely to result in the increase of other yet unknown recessively inherited defects.

In conclusion, we identified the *RAPGEF6*:c.1793_1794ins36 variant leading to a splice defect in the *RAPGEF6* gene as candidate causative variant for LP in Miniature Bull Terriers. The variant represents a major genetic risk factor for a complex trait. The genotype at this variant is not perfectly associated with the phenotype indicating heterogeneity and/or the presence of additional modifier genes and/or environmental risk factors. The molecular pathogenesis of LP remains unclear. Our data facilitate genetic testing of Miniature Bull Terriers and Bull Terriers to prevent the non-intentional breeding of LP affected dogs. LP affected dogs may serve as models to further clarify the elusive physiological role of RAPGEF6 *in vivo*.

## Materials and methods

### Ethics statement

All animal experiments were performed according to the local regulations. The dogs in this study were examined with the consent of their owners. The collection of blood samples was approved by the “Cantonal Committee For Animal Experiments” (Canton of Bern; permit 75/16).

### Animals and samples

Bull Terriers with their characteristic egg-shaped head were founded as a dog breed in the 1850s in the United Kingdom. Originally, there were no size standards in this breed and smaller dogs were bred as a variety of the regular Bull Terrier. Eventually, two sub-populations formed and the Miniature Bull Terrier with a maximum height of 35.5 cm was recognized as an independent breed in 1991 by the American Kennel Club (AKC) and in 2011 by the European Fédération Cynologique Internationale (FCI). Therefore, Bull Terriers and Miniature Bull Terriers share a common ancestral gene pool, but represent independent closed populations today.

This study included samples from 385 Miniature Bull Terriers (43 cases / 200 controls / 38 unknown phenotype / 104 excluded). The study also included 75 Bull Terriers (74 unknown phenotype / 1 excluded), 28 American Staffordshire Terriers (19 cases / 9 controls), 90 French Bulldogs (3 with impaired laryngeal function / 8 controls / 79 unknown phenotype), 12 Pugs (1 with impaired laryngeal function / 2 controls / 9 unknown phenotype), 10 Staffordshire Bull Terriers (4 cases / 6 controls) and >1000 dogs from many different breeds, which were assumed to be free of early-onset LP (Table 1, S1 Table, S5 Table, S6 Table). EDTA blood samples were taken for DNA isolation. PAXgene blood tubes (Qiagen) were used to collect blood samples for RNA isolation.

### Phenotype assignment

Case / control status was mostly based on owner’s report without endoscopic assessment of the upper airways. Controls: Dogs age two years and older with no reported breathing difficulties or diagnosed endocrinopathies were designated as controls. In three dogs, physiologic laryngeal function had been endoscopically confirmed. Cases: Dogs with breathing difficulties reported by the owner were designated as cases. Dogs with an onset of clinical signs older than 8 years and dogs suffering from hypothyroidism were excluded from association analyses and classified as unknown regarding the LP phenotype. In 33 Miniature Bull Terriers with breathing problems, laryngeal dysfunction had been endoscopically confirmed (S1 Table).

### Laryngoscopy

Endoscopy of the upper airways was performed in three different institutions to assess laryngeal function. The examination was consented by the owners. Dogs were anesthetized with randomly assigned anesthetic protocols. Dogs were positioned in sternal recumbency and the head was elevated to the level of physiologic carriage by using e.g. a maxillary sling. The mouth was opened and the larynx evaluated during spontaneous breathing with rigid straight 0°-endoscopes of different diameter (Karl Storz, Germany). In two institutions breathing was additionally stimulated intravenously with doxapram hydrochloride (1–2 mg/kg body weight) [4,35–37]. According to Gross et al. [37] laryngeal function was characterized as either normal with visible abduction during inspiration or abnormal, without abduction during inspiration, and resulting in the diagnosis of LP.

### DNA isolation and SNV genotyping

We isolated genomic DNA from EDTA blood samples. Eighty-five dogs were genotyped for 220,853 SNVs on the illumina canine_HD chip (S1 File).

### GWAS

A set of 23 cases and 62 controls was selected for the GWAS. GWAS was done using a mixed model in RStudio with the GenABEL package. A polygenic model of the hglm package [38], with a kinship matrix based on autosomal markers in the cleaned dataset as random effect, was estimated and a score test for association using the function “mmscore” was performed. We corrected for multiple testing using Bonferroni correction with a significance level of 0.05. QQ plots were created using qqman version 0.1.4 [39].

### Phasing and haplotype analysis

We inferred haplotype phase using the program fastPHASE version 1.4.0 [40]. We phased chromosome 11 using all cases and control dogs together in a single run. We visually inspected the phased haplotypes for the region of interest on chromosome 11 using Excel for shared haplotype blocks among cases. Recombination events on either side of the shared disease-associated haplotype defined the borders of the critical interval.

### Whole genome sequencing of an LP affected Miniature Bull Terrier

An Illumina PCR-free TruSeq fragment library with 400 bp insert size of an LP affected Miniature Bull Terrier (MB003) was prepared. We collected 123 million 2 x 150 bp read-pairs or 12.5 x coverage on a HiSeq3000 instrument. The reads were mapped to the dog reference genome assembly CanFam3.1 and aligned using Burrows-Wheeler Aligner (BWA) version 0.7.5a [41] with default settings. The generated SAM file was converted to a BAM file and the reads were sorted by coordinate using samtools [42]. Picard tools (http://sourceforge.net/projects/picard/) was used to mark PCR duplicates. To perform local realignments and to produce a cleaned BAM file, we used the Genome Analysis Tool Kit (GATK version 2.4.9, 50) [43]. GATK was also used for base quality recalibration with canine dbSNP version 139 data as training set. The sequence data were deposited under the study accession PRJEB16012 and sample accession SAMEA4867920 at the European Nucleotide Archive.

### Variant calling

Putative SNVs were identified and annotated in each of 602 whole genome sequences as described [44]. For the filtering of candidate causative variants in the case, we excluded 1 Miniature Bull Terrier and 2 Bull Terriers without LP phenotype information. Thus, we used the case genome and 598 control genomes, which were either publicly available [45] or produced during other projects of our group or contributed by members of the Dog Biomedical Variant Database Consortium [44]. A detailed list of these control genomes is given in S2 Table.

### Gene analysis

We used dog CanFam 3.1 reference genome assembly together with the NCBI annotation release 105 for all analyses. Numbering within the canine *RAPGEF6* gene corresponds to the accessions XM_846793.5 (mRNA) and XP_851886.2 (protein).

### Sanger sequencing and targeted genotyping

We used Sanger sequencing to confirm the *RAPGEF6*:c.1793_1794ins36 candidate variant. A 265 bp or 301 bp fragment containing the variable position was PCR amplified (35 cycles) from genomic DNA using AmpliTaq Gold 360 Master Mix (ThermoFisher). Primers are given in the S7 Table. After treatment with shrimp alkaline phosphatase and endonuclease I, PCR products were directly sequenced on an ABI 3730 capillary sequencer (ThermoFisher). We analyzed the Sanger sequence data using the software Sequencher 5.1 (GeneCodes). To genotype larger numbers of samples, we performed fragment size analysis on a Fragment Analyzer capillary gel electrophoresis instrument (Advance Analytical).

### RNA isolation and RT-PCR

Total RNA was extracted from blood samples using the PAXgene Blood RNA Kit IVD (Qiagen). Polyadenylated mRNA was reverse transcribed into cDNA using the SuperScript IV Reverse Transcriptase Kit (ThermoFisher) with oligo d(T) primers. Primer sequences for the PCR on the synthesized cDNA are listed in the S4 Table. The products were analyzed on a Fragment Analyzer capillary gel electrophoresis instrument (Advanced Analytical). The sequence of the obtained RT-PCR products was confirmed by Sanger sequencing as described above.

### Relative risk

Relative risk (RR) for Miniature Bull Terriers was calculated by using the MedCalc online statistical tool. Based on the existing information, dogs were classified in eight groups (case, endoscopy confirmed; case, owner reported; control, endoscopy confirmed; control, owner reported; excluded, case ≥ 8 years age of onset; excluded, control < 2 years of age; excluded, hypothyroidism; phenotype unknown). All phenotype and genotype information used for the calculation are listed in S1 Table.

### *ADAMTS3* genotyping

We used Sanger sequencing to genotype the dogs for the *ADAMTS3*:c.2786G>A variant. A 230 bp fragment containing the variable position was PCR amplified from genomic DNA using AmpliTaq Gold 360 Master Mix (ThermoFisher). Primers are given in the S7 Table. After treatment with shrimp alkaline phosphatase and endonuclease I, PCR products were directly sequenced on an ABI 3730 capillary sequencer (ThermoFisher). We analyzed the Sanger sequence data using the software Sequencher 5.1 (GeneCodes).

## Supporting information

S1 File SNV genotypes of 85 Miniature Bull Terriers. (ZIP)

S1 Table Phenotype and genotype information of all Miniature Bull Terriers included in this study. (XLSX)

S2 Table Sample designations, breed information and accessions on 602 dogs or wolves with genome sequences. (XLSX)

S3 Table Private variants in the genome of an affected Miniature Bull Terrier. (XLSX)

S4 Table Structural variants in the LP affected Miniature Bull Terrier. (XLSX)

S5 Table Phenotype and genotype information of 5 other breeds. (XLSX)

S6 Table *RAPGEF6*:c.1793_1794ins36 genotypes of 541 dogs from 69 different dog breeds. (XLSX)

S7 Table Primer sequences. (XLSX)
